# ﻿First record of the subfamily Geracinae Brindle, 1971 and genus *Pseudovostox* Borelli, 1926 (Dermaptera, Spongiphoridae) in China, with a description of a new species

**DOI:** 10.3897/zookeys.1254.162412

**Published:** 2025-10-08

**Authors:** Zhi-Teng Chen, Chao Jiang

**Affiliations:** 1 School of Grain Science and Technology, Jiangsu University of Science and Technology, Zhenjiang 212004, Jiangsu Province, China Jiangsu University of Science and Technolog Zhenjiang China; 2 State Key Laboratory for Quality Ensurance and Sustainable Use of Dao-di Herbs, National Resource Center for Chinese Materia Medica, China Academy of Chinese Medical Sciences, Beijing 100700, China China Academy of Chinese Medical Sciences Beijing China

**Keywords:** Biodiversity, earwig, Geracinae, identification key, morphology, new record, taxonomy

## Abstract

The subfamily Geracinae Brindle, 1971, and the genus *Pseudovostox* Borelli, 1926, are recorded for the first time from China. These new records were confirmed by collections from Guiyang City in Guizhou Province, located in southwestern China. *Pseudovostox guizhouensis***sp. nov.** is described and illustrated based on specimens from this region. Although the new species closely resembles the Oriental species *Pseudovostox fasciatus* (de Bormans, 1894), it can be distinguished by a combination of unique morphological characters. An updated key and distribution map for *Pseudovostox* species are provided to aid future research and species identification.

## ﻿Introduction

The family Spongiphoridae is a diverse and ecologically important group of earwigs (Dermaptera) mainly found in tropical and subtropical regions. Currently, the family is classified into 13 subfamilies, comprising 42 genera and 527 species (Hopkins et al. 2025). Among these, the subfamily Geracinae Brindle, 1971 is relatively diverse, containing six genera and 29 species distributed across South Asia and East Africa (Hopkins et al. 2025). Species of Geracinae are generally small to very small (body length shorter than 10 mm) and possess distinctive morphological traits such as tarsal arolia and forceps of similar shape in both males and females (Brindle 1971).

*Pseudovostox* Borelli, 1926 is the most species-rich genus within Geracinae, containing 13 species found in both the Oriental and African regions (Steinmann 1990; Srivastava 2003). Species within this genus exhibit considerable morphological diversity, with variations in body coloration, penultimate sternite structure, and genitalia shape, all of which are essential for species identification and taxonomic classification. Despite the diversity, the distribution of *Pseudovostox* has been underexplored, particularly in East and Southeast Asia.

Prior to this study, neither members of Geracinae nor *Pseudovostox* had been reported from China. This gap in the taxonomic record prompted an investigation into the Dermaptera fauna of Guiyang City, located in Guizhou Province, southwestern China. The region is characterized by its diverse ecological conditions, which likely support a variety of earwig species. In June of both 2024 and 2025, a series of earwig specimens were collected from Guiyang City. Initial morphological assessments indicated significant similarities with *Pseudovostox fasciatus* (de Bormans, 1894), a species previously recorded from Myanmar, Thailand, and Vietnam (Steinmann 1990; Srivastava 2003). However, further examination of the specimens revealed several key morphological differences, suggesting the presence of an undescribed species. In this paper, we describe and illustrate this new species, *Pseudovostox guizhouensis* sp. nov., which represents the first formal record of both Geracinae and *Pseudovostox* in China. An updated key for the identification of *Pseudovostox* species is also provided to support future research on the genus and its distribution.

## ﻿Material and methods

The specimens were collected from different areas of Guiyang City, Guizhou Province, China in June of both 2024 and 2025 and preserved in 75% alcohol. The type specimens of this new species are deposited in the Insect Collection of Jiangsu University of Science and Technology (**ICJUST**), Jiangsu Province, China. Morphological examinations and identifications were conducted using an SDPTOP SZM45 stereomicroscope. Images were captured with a Canon EOS 5DSR camera with a Canon MP-E 2.8/65 mm macro lens. Plates were prepared using the software Adobe Photoshop 2024. The morphological terminology follows Steinmann (1990).

## ﻿Results

### ﻿ Pseudovostox guizhouensis sp. nov.

Taxon classificationAnimaliaDermapteraSpongiphoridae

AE3B3C84-EFA7-55B0-8545-AD56BF0DFB41

https://zoobank.org/97DAC96D-D9BE-4083-BFD7-EFD0AD4CBDEF

**Figure 1.**
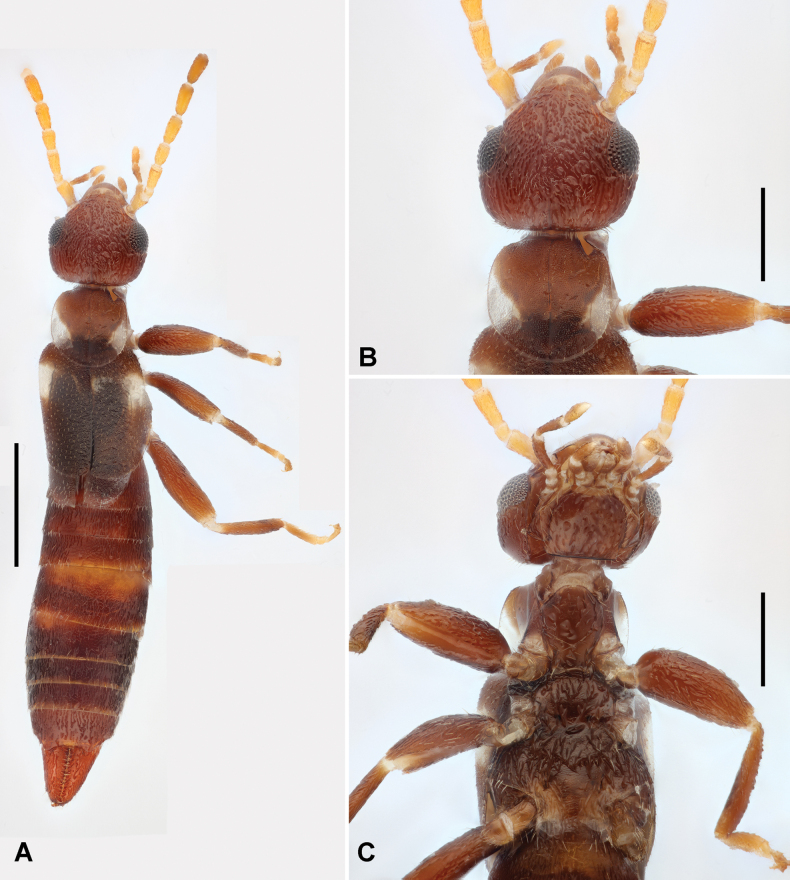
*Pseudovostox guizhouensis* sp. nov., male holotype. A. Habitus, dorsal view; B. Head and pronotum, dorsal view; C. Head and thorax, ventral view. Scale bars: 1 mm (A); 0.5 mm (B, C).

**Figure 2.**
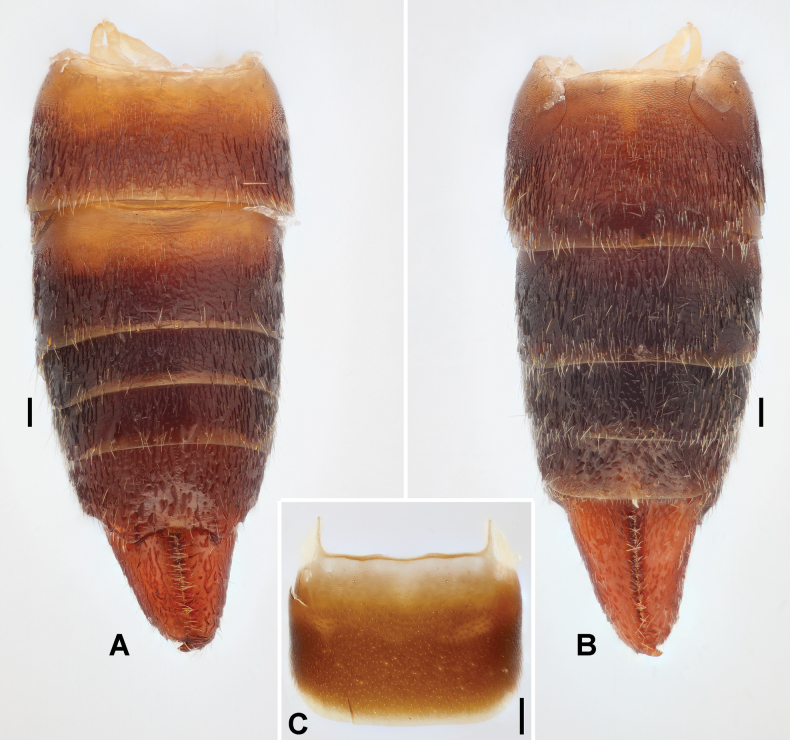
*Pseudovostox guizhouensis* sp. nov., male holotype. A. Abdomen, dorsal view; B. Abdomen, ventral view; C. Penultimate sternite, ventral view. Scale bars: 0.1 mm.

**Figure 3.**
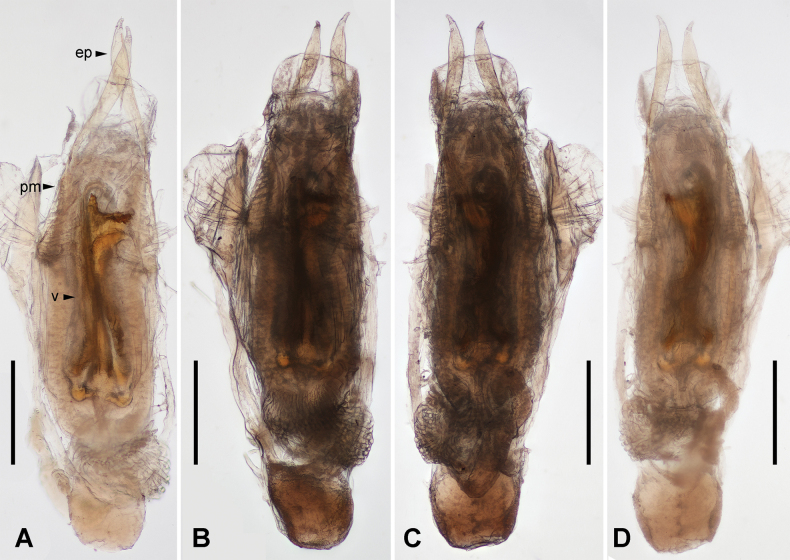
*Pseudovostox guizhouensis* sp. nov., male holotype. A. Genitalia under reflected light, dorsal view; B. Genitalia under transmitted light, dorsal view; C. Genitalia under transmitted light, ventral view; D. Genitalia under reflected light, ventral view. Abbreviations: ep – external paramere; pm – paramere; v – virga. Scale bars: 0.5 mm.

**Figure 4.**
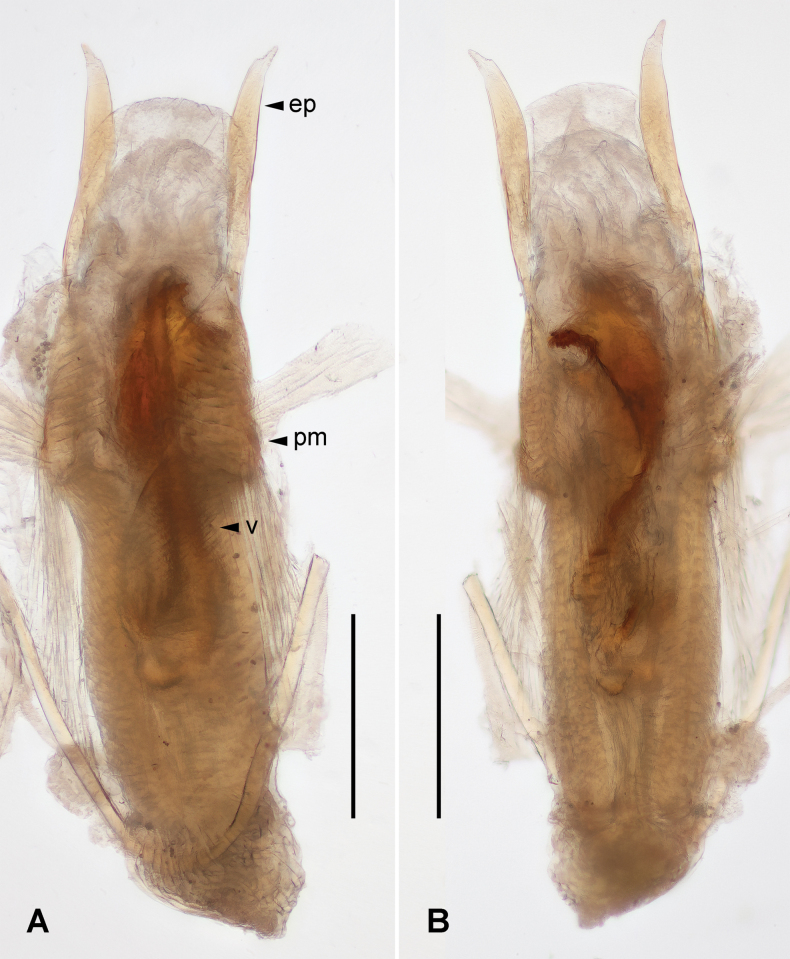
*Pseudovostox guizhouensis* sp. nov., male paratype. A. Genitalia under reflected light, dorsal view; B. Genitalia under reflected light, ventral view. Abbreviations: ep – external paramere; pm – paramere; v – virga. Scale bars: 0.5 mm.

**Figure 5.**
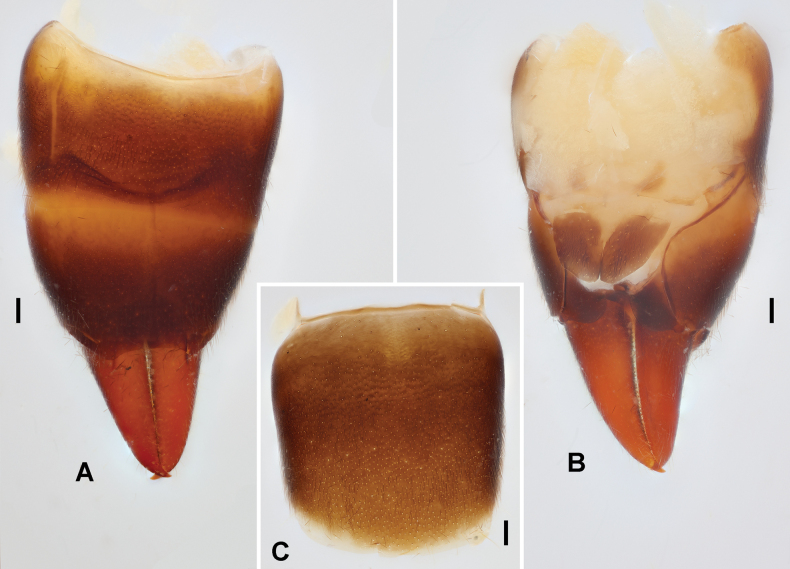
*Pseudovostox guizhouensis* sp. nov., female paratype A. Abdomen, dorsal view; B. Abdomen, ventral view; C. Penultimate sternite, ventral view. Scale bars: 0.1 mm.

**Figure 6.**
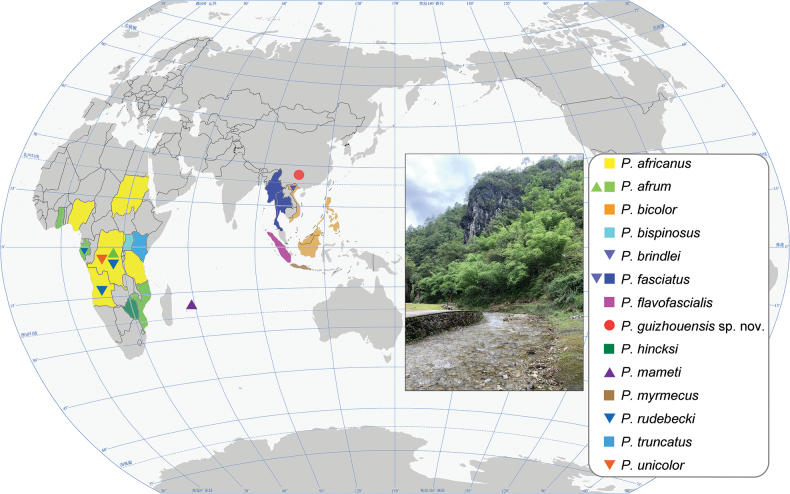
General distribution of *Pseudovostox* species. Inset: collecting environment in Guizhou Province, China. Different color and symbols represent different areas: *P. africanus* (Angola, Nigeria, Congo, Sudan, Tanzania), *P. afrum* (Congo, Gabon, Ghana, Mozambique), *P. bicolor* (Borneo, Philippine Islands, Vietnam), *P. bispinosus* (Uganda), *P. brindlei* (Vietnam), *P. fasciatus* (Myanmar, Thailand, Vietnam), *P. flavofascialis* (Sumatra), *P. guizhouensis* sp. nov. (Guizhou Province of China), *P. hincksi* (Zimbabwe), *P. mameti* (Mauritius), *P. myrmecus* (Java), *P. rudebecki* (Angola, Congo, Gabon), *P. truncatus* (Kenya), *P. unicolor* (Congo).

Figs 1, 2, 3, 4, 5, 6

#### Type material.

***Holotype***: China • ♂; Guizhou Province, Guiyang City, Xiangzhigou; 26.7775°N, 106.9346°E; 1,151 m; 17.vi.2024; Zhi-Teng Chen leg. ***Paratypes***: China • 7♂♂16♀♀; same data as holotype; 7♂♂2♀♀, Guizhou Province, Guiyang City, Herang; 26.7270°N, 106.8766°E; 1,010 m; 18.vi.2024; Zhi-Teng Chen leg. • 1♂, Guizhou Province, Guiyang City, Xiaba Town, near Baishui River; 26.7254°N, 106.9313°E; 970 m; 18.vi.2024; Zhi-Teng Chen leg; 11♂♂15♀♀, Guizhou Province, Guiyang City, Xiangzhigou; 26.7768°N, 106.9347°E; 1,130 m; 26.vi.2025; Zhi-Teng Chen, Qing Li leg.

#### Differential diagnosis.

The new species shares a similar pale transverse stripe on the tegmina with *P. fasciatus* (Steinmann 1990; Srivastava 2003). However, it can be distinguished by several morphological traits (Steinmann 1990; Srivastava 2003): the male head is longer than wide (vs. wider than long in *P. fasciatus*), the pronotum is long with a rounded anterior margin (vs. transverse with a truncate anterior margin), and the pale stripe on the tegmina is near one-fourth of its length (vs. near one-eighth in *P. fasciatus*). Additionally, the male’s penultimate sternite of the new species has a mostly truncate posterior margin (vs. concave in *P. fasciatus*), and the external paramere is widened subapically and constricted apically with an obtuse apex (vs. gradually tapering to an acute apex in *P. fasciatus*). The female of the new species also differs from that of *P. fasciatus* in having posterior processes on the ultimate tergite and straight inner forceps margins, whereas the female of *P. fasciatus* lacks such processes, and the inner margin of the forceps is sinuate (Brindle 1973a; Steinmann 1990). An updated key to species of *Pseudovostox* based on Steinmann (1990) is provided below for species delimitation.

#### Description.

**Male. *Length*.** Body length (excluding forceps) 4.5–5 mm, forceps length 0.5–0.8 mm.

***Coloration*.** Head and pronotum dark brown, latter with white posterolateral sides (Fig. 1A–C); antennae yellow at base, pale brown apically. Tegmina dark brown, with a pale transverse stripe near shoulder, the stripe gradually narrowed mediad (Fig. 1A). Scales of hind wings entirely white. Abdomen dark brown; legs and forceps brown (Fig. 1A).

***Head*.** Head longer than wide, tumid; postfrontal and coronal sutures indistinct; hind margin truncate (Fig. 1A–C). Eyes slightly shorter than post-ocular area. Antenna 12-segmented; basal segment about half the length of distance between antennal bases; second segment as long as wide; third segment three times as long as wide; fourth segment two times as long as wide; fifth and subsequent segments slightly longer than third.

***Thorax*.** Pronotum as broad as long, slightly widened posteriorly; anterior margin slightly rounded, lateral and posterior margins rounded; prozona convex and metazona depressed; median sulcus distinct (Fig. 1A–C). Tegmina well-developed, twice as long as pronotum, pubescent and impunctate; scales of hind wings near as long as one-third length of pronotum (Fig. 1A). Legs with strong femora, thin tibiae and tarsi, and developed tarsal arolia between claws; hind tarsi with first segment 1.5 times longer than combined length of segments 2–3, second segment near as long as wide, third segment twice as long as second (Fig. 1A).

***Abdomen*.** Abdomen fusiform, convex, widest at segments 6–7, cuticle pubescent and impunctate (Fig. 1A). Ultimate tergite transverse, sloping and narrowed posteriad; posterior margin concave medially, with subtriangular processes above base of each branch of forceps (Fig. 2A). Forceps short, contiguous, pubescent, mostly straight, upcurved, apically tapering and gently hooked mediad; inner margins denticulate (Fig. 2A, B). Penultimate sternite transverse, 1.5 times wider than long, posterior margin mostly truncate; basolateral projections short, near one-fifth of sternal length (Fig. 2C).

***Genitalia*.** Genitalia slender (Fig. 3A–D); paramere narrowed basally, widened medially, with rounded anterior margin; external paramere slender, widened subapically, apex narrowed, obtuse; virga thick; inner structure complicated, with two semicircular basal sclerites and a toothed apical sclerite. Genitalia of male paratype slightly varied in shape of external parameral apex and inner sclerite (Fig. 4A, B).

**Female.** Body length without forceps 4.5–6 mm, length of forceps 0.5–0.8 mm. Body structures and coloration identical to male holotype; tergites and sternites longer than male (Fig. 5A, B). Penultimate sternite subquadrate, posterior margin rounded; basolateral projections very short, about 1/11 of sternal length (Fig. 5C). Genitalia with two dark, oval sclerites (Fig. 5B).

#### Etymology.

The new species is named after Guizhou Province, the type locality.

#### Distribution.

The species is currently known only from Guizhou Province of southwest China (Fig. 6).

#### Biology.

**Figure 7.**
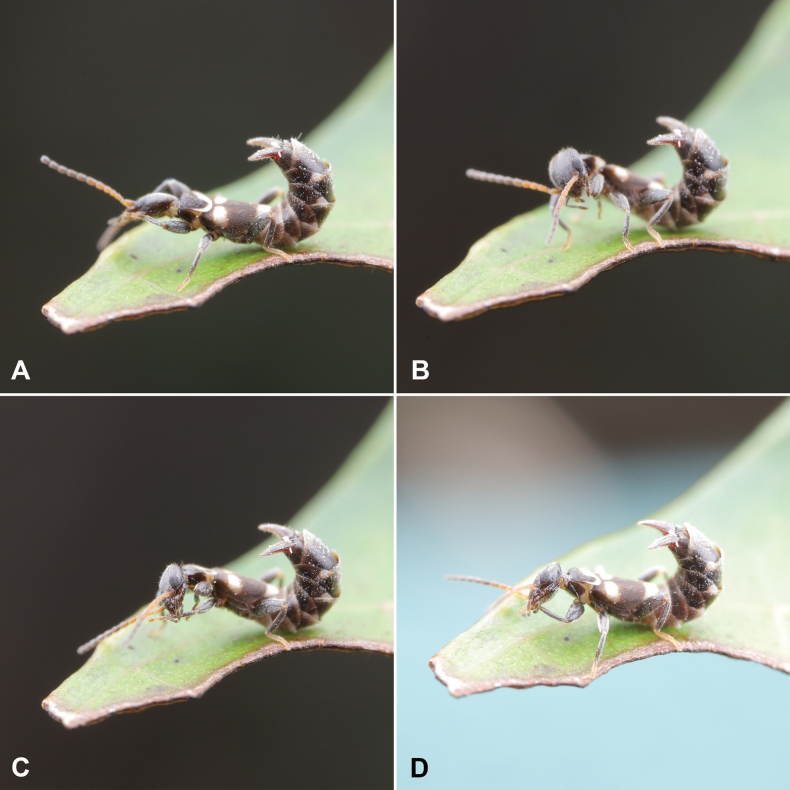
Living habitus of *Pseudovostox guizhouensis* sp. nov. A, B. Defensive posture; C, D. Leg-cleaning behavior. Photos taken by Dr Ri-Xin Jiang (Guizhou University, China).

Specimens were collected from riparian vegetation using a sweeping net (Fig. 6). This species exhibited no specific vegetation preference and was collected from leaves of bamboo, gramineous weeds, tea trees, vegetables and various other plants. It occurred in high densities near the water’s edge. When disturbed, individuals displayed a typical defensive posture by raising their forceps, a behaviour similar to that of other earwig species (Fig. 7A, B). In addition, they exhibited leg-cleaning behavior (Fig. 7C, D). During a second collecting trip to Guizhou Province in 2025, specimens were encountered under rainy conditions. Individuals immediately became immobile when they contacted the water droplets on the inner surface of the collecting bottle. After several hours, once the water had evaporated, the specimens became very active again. This response suggests that the new species may exhibit thanatosis-like behavior.

### ﻿Updated key to species of *Pseudovostox* based on males

**Table:** 

1	Oriental distribution	**2**
–	African distribution	**7**
2	Eyes shorter than length of head behind eyes	**3**
–	Eyes longer than length of head behind eye	**4**
3	Head and pronotum longer than wide; pale stripe of tegmina near one-fourth of tegmina length	***P. guizhouensis* sp. nov.**
–	Head and pronotum wider than long; pale stripe of tegmina near 1/8 of tegmina length	***P. fasciatus* (de Bormans, 1894)**
4	Tegmina and hind wings unicolorous	***P. bicolor* Borelli, 1926**
–	Tegmina with contrasting colors	**5**
5	Hind wings absent	***P. myrmecus* (Burr, 1908)**
–	Hind wings present	**6**
6	Pronotum with yellow lateral and posterior margins; tegmina with yellow transverse stripe near shoulder and posterior margin	***P. brindlei* Srivastava, 2003**
–	Pronotum broadly yellow posteriorly; tegmina with an anteromedial yellow patch	***P. flavofascialis* Brindle, 1973**
7	Tegmina entirely or basally yellow	**8**
–	Tegmina unicolorous, dark brown or blackish	**10**
8	Pronotum, tegmina, and hind wings unicolourous, yellowish-brown	***P. unicolor* Brindle, 1970**
–	Pronotum or tegmina at least partially darkened	**9**
9	Hind wings blackish; male penultimate sternite with a small triangular posterior excision	***P. hincksi* Brindle, 1970**
–	Hind wings yellow; male penultimate sternite with broadly concave posterior margin	***P. africanus* (Brindle, 1968)**
10	Male penultimate sternite with almost truncate posterior margin	***P. truncatus* Brindle, 1970**
–	Male penultimate sternite with posterior margin not truncate	**11**
11	Male penultimate sternite with posterior margin produced with two distal spines	***P. bispinosus* Brindle, 1970**
–	Male penultimate sternite without posterior spines	**12**
12	Hind wings absent	***P. mameti* (Hincks, 1950)**
–	Hind wings present	**13**
13	Pronotum as long as wide; male penultimate sternite with a narrow posterior excision	***P. rudebecki* Brindle, 1969**
–	Pronotum longer than wide; male penultimate sternite with a wide posterior excision	***P. afrum* Menozzi, 1935**

## ﻿Discussion

The species of *Pseudovostox* are primarily distinguished by a combination of morphological features, including eye length, head and pronotum shape, color pattern on pronotum and tegmina, hind wing configuration, penultimate sternite shape, and genitalia structure (Brindle 1973b; Steinmann 1990; Srivastava 2003). Species of this genus have traditionally been divided into two distinct geographical groups: the Oriental group and the African group (Steinmann 1990). The Oriental group includes species from Southeast Asia, while the African group comprises species found throughout the African continent (Fig. 6). Although there is some overlap in species distributions within each group, the distinction remains useful for understanding their broader biogeography. Steinmann (1990) placed *Pseudovostox mameti* (Hincks, 1950) from Mauritius in the Oriental group; however, it is more suitable to put it in the African group. This highlights the complexity of species boundaries within *Pseudovostox* and the need for continued taxonomic revisions as new specimens and distribution data become available.

The discovery of *Pseudovostox* in Guizhou Province significantly extends the known distribution of the genus into southwestern China, a region that has remained largely unexplored in terms of its Dermaptera fauna. The morphological differences between *P. guizhouensis* sp. nov. and its closely related species, particularly in the head, pronotum, penultimate sternite, and genitalia, are considerable. These differences not only justify the description of a new species but also highlight the morphological diversity within *Pseudovostox*. This new species adds to our understanding of how *Pseudovostox* can adapt to diverse ecological conditions across its distribution. Furthermore, this discovery underscores the importance of exploring understudied regions, such as southwestern China, which are likely to yield additional new species, enriching our knowledge of *Pseudovostox* and the subfamily Geracinae.

In conclusion, the finding of *P. guizhouensis* sp. nov. represents a significant addition to the taxonomic knowledge of Spongiphoridae in China and provides new insights into the distribution and diversity of the genus *Pseudovostox*. This discovery highlights the potential for further exploration in under-studied regions of Asia and contributes to a more comprehensive understanding of the family’s global biodiversity.

## Supplementary Material

XML Treatment for Pseudovostox guizhouensis

## ﻿Additional information

### Use of AI

No use of AI was reported.

### Author ORCIDs

Zhi-Teng Chen https://orcid.org/0000-0002-6331-8978

Chao Jiang https://orcid.org/0000-0003-1841-1169

### Data availability

All of the data that support the findings of this study are available in the main text.
